# Cytotoxic activity of bimetallic Ag@Se green synthesized nanoparticles using Jerusalem Thorn (*Parkinsonia aculeata*)

**DOI:** 10.3389/fchem.2024.1343506

**Published:** 2024-03-25

**Authors:** Hanaa A. Hassanin, Amel Taha, Hairul-Islam Mohamed Ibrahim, Emad A. Ahmed, Hisham Mohamed, Hoda Ahmed

**Affiliations:** ^1^ Department of Chemistry, Faculty of Science, Ain Shams University, Cairo, Egypt; ^2^ Department of Chemistry, College of Science, King Faisal University, Hufof, Saudi Arabia; ^3^ Department of Chemistry, Faculty of Science and Technology, Al-Neelain University, Khartoum, Sudan; ^4^ Biological Sciences Department, College of Science, King Faisal University, Al-Ahsa, Saudi Arabia; ^5^ Division of Microbiology and Immunology, Pondicherry Centre for Biological Sciences and Educational Trust, Pondicherry, India; ^6^ Lab of Molecular Physiology, Department of Zoology, Faculty of Science, Assiut University, Asyut, Egypt; ^7^ Date Palm Research Center of Excellence, King Faisal University, Hufof, Saudi Arabia; ^8^ Agricultural Research Center, Ministry of Agricultural, Giza, Egypt; ^9^ Botany and Microbiology Department, Faculty of Science, Alexandria University, Alexandria, Egypt

**Keywords:** *Parkinsonia aculeata L*, Ag@Se nanoparticles, RAW 264.7, interleukin-1β, interleukin-6, phosphoinositide 3 kinase, bimetallic and nuclear factor kappa B, cytotoxicity

## Abstract

**Introduction:** The process of green synthesis of metal nanoparticles is considered to be eco-friendly and cost-effective.

**Methods:** In this study, bimetallic Ag@Se-P and Ag@Se-S nanoparticles were synthesized successfully using *Parkinsonia aculeata* aerial parts and seed extracts. The phytochemical contents in *P. aculeata* aerial parts and seed aqueous extract serve as reducing and stabilizing capping agents without the need for any chemical stabilization additive in the synthesis of bimetallic nanoparticles.

**Result and Discussion:** The obtained results from UV-vis spectrophotometry, scanning electron microscopy (SEM), X-ray powder diffraction (XRD), energy-dispersive X-ray spectroscopy (EDS), transmission electron microscopy (TEM), and Fourier-transform infrared spectroscopy (FT-IR) confirmed the successful synthesis of bimetallic nanoparticles with cluster irregular spherical morphology, crystalline nature, and average particle sizes of 17.65 and 24.36 nm for Ag@Se-S and Ag@Se-P, respectively. The cytotoxicity assessment of greenly synthesized nanomaterials using seed and plant extracts showed cell inhibition >50 μg/mL. Ag@Se-S and Ag@Se-P seed and plant extracts significantly reduced LPS-induced inflammation, which was assessed by NO and cytokines IL-1β, IL-6, and TNF-α. The mRNA and protein expression levels of phosphoinositide 3 kinase (PI3K) and nuclear factor kappa B (NFkB) were significantly overexpressed in LPS-induced RAW 264.7 cell lines. Ag@Se-S and Ag@Se-P downregulated the expression of PI3K and NFkB in LPS-induced cell models.

## Introduction

Nanotechnology research provides significant advancements in the production of materials and nanoelectronics. This type of research has a wide range of applications in various fields, including biotechnology, energy, healthcare, environment, and medicine (Yaqoob et al., 2020a; Hassanin and Taha, 2022; Taha and Hassanin, 2022). The unique chemical and physical properties of nanomaterials were found to be size-dependent. However, they possess high optical, magnetic, and electrical properties and also have high thermal and mechanical stability (Sumbal et al., 2019). Nano-sized alloys are inorganic nanoparticles that may contain more than one metal and are called bimetallic nanoparticles. Bimetallic nanoparticles have been extensively investigated due to their substantial potential in technological and biological fields. Due to their photothermal and optical properties, the noble metal nanoparticles (Ag, Au, and Pt) have also received great attention (Yaqoob et al., 2020b). Noble metal nanoparticles have a wide range of biomedical applications, such as gene delivery, thermal ablation, radiotherapy enhancement, and drug delivery (Yamada et al., 2015). Silver (Ag) nanoparticles are considered the most widely used nanomaterials in various applications due to their outstanding properties, including electrical and optical properties, making them suitable for use in medical implants, surgical equipment, wound healing, cosmetics, food handling, packaging devices, and water purifiers (Biswas and Dey, 2015; Hassanin et al., 2021). However, studies have revealed that noble metal nanoparticles are toxic to normal cells at higher concentrations. Therefore, it is important to look for new materials with biological activities that are nontoxic to normal cells. Selenium (Se), an essential trace component of selenoproteins and enzymes, was reported to have antioxidant properties that help break down peroxides. Due to their protective properties, Se nanoparticles have been widely used as safe antimicrobial, anticancer, and therapeutic agents (Maiyo and Singh, 2017; Nguyen et al., 2017). However, considering the wide variety of biological applications of Ag and Se nanoparticles, a nanocomposite containing both Ag and Se could be a viable material for different biological applications.

The synthesis of bimetallic nanoparticles has been performed via chemical reduction or green synthesis (Ahmad et al., 2020). However, only few studies have reported the green synthesis of Ag–Se NPs (Kumar et al., 2012; Sibiya and Moloto, 2017; García et al., 2020). The synthesis of nanoparticles by chemical methods is more common because it produces a high yield and gives good control over the size and morphology of nanoparticles. However, the excessive use of chemicals has some drawbacks, such as toxicity to the environment and high costs. The green synthesis of nanoparticles using plant extracts has been extensively applied recently since most plant extracts contain biologically active components that might increase the biological activity of the manufactured nanoparticles (Hassanin et al., 2021; Hassanin and Taha, 2022; Taha and Hassanin, 2022).

*Parkinsonia aculeata L.* (Fabaceae) is an evergreen, spiny shrub or tree with compound bipinnate leaves and brightly yellow-scented flowers growing in tropical and subtropical regions as an ornamental plant. In folk medicine, its leaves and flowers are used for rheumatism treatment, while leaves, fruits, and stems are used to cure malaria and fever. It has immense amounts of medicinal compounds, including carbohydrates, saponins, flavonoids, alkaloids, and essential oils (López-Millán et al., 2019; Abdelaziz et al., 2020). In addition, several studies proved the possible applications of leaf, stem, and flower extracts as antibacterial, anticancer, antispermatogenic, and antioxidants (Shehu et al., 2018; Das and Latha, 2022).

This study represents the inaugural utilization of *P. aculeata* extracts of both whole plants and seeds used as reducing, capping, and stabilizing agents for the biogenic synthesis of bimetallic Ag@Se nanoparticles, a novel approach that diverges from its conventional applications. The introduction of this plant in the synthesis process is distinctive, potentially unveiling previously unidentified properties or compounds that could significantly contribute to advancements in the field of nanotechnology. This innovative application holds promise for future developments in medicinal and material sciences, particularly in enhancing the bioactivity of nanoparticles using *Parkinsonia.* The manufactured Ag@Se-P (synthesized using plant extracts) and Ag@Se-S (synthesized using seed extracts) samples were characterized using various analytical techniques. The biological activity of plant extracts, seed extracts, and synthesized materials was evaluated for antioxidant, cytotoxic, scratch wound healing, and anti-inflammatory activities.

## Materials and methods

### Materials and chemicals

The vegetative aerial parts of *P. aculeata L.* were collected from the Hofuf region, Al-Ahsa, Eastern Province, Saudi Arabia, in January 2022. The plants were washed twice with tap water, followed by distilled water, and then left in the shade to dry. The seeds of *P. aculeata* L. were collected from the same trees in June 2022. Both plants and seeds were ground into a fine powder using a Wiley mill grinder.

Silver nitrate (AgNO_3_, ≥98%) and sodium selenite (Na_2_SeO_3_, ≥98%) were purchased from Sigma-Aldrich. Trichloroacetic acid (TCA), 3-(4,5-dimethyl thiazol-2-yl)-2,5-diphenyl tetrazolium bromide (MTT), phosphate-buffered saline (PBS), and gallic acid were supplied by Merck (Darmstadt, Germany). Folin–Ciocalteu reagent, 2,2-diphenyl-1-picrylhydrazyl (DPPH), potassium ferricyanide, and iron (III) chloride were obtained from Sigma-Aldrich Chemical Co. (St Louis, MO, USA). Eagle’s minimum essential medium (EMEM), fetal bovine serum (FBS), trypsin-0.5%, and antibiotics (penicillin (5,000 units/mL)/streptomycin (5,000 μg/mL)) were purchased from Lonza BioWhittaker (Verviers, Belgium). The macrophage cells (RAW 264.7) were obtained from PCBS, Pondicherry, India. Sterile plasticware for cell culture was purchased from Corning Inc. (New York, NY, USA). All other reagents used in this work are of laboratory grade. Ultrapure (18 MOhm) water (Millipore, France) was used throughout the experiments.

### Preparation of plant extract

For sample preparation, 4 grams of air-dried powdered (20 µm) *P. aculeata* leaves (PALs) and *P. aculeata* seeds (PASs) were each soaked in 100 mL of double-distilled water at 80°C for 30 min. The extract was let to cool down, filtrated using Whatman filter paper No. 1, and then centrifuged at 6,000 rpm/5°C to get a clear aqueous extract.

### Total phenol estimation of plant leaf and seed extracts

Total phenols of both *P. aculeata* leaves and seeds were assessed according to the Singleton and Rossi method (Singleton and Joseph, 1965). Folin–Ciocalteu (500 μL) and 6 mL of distilled water were added to 1 mL of each extract, and the mixture was vortexed for 5 min. Afterward, 1.5 mL of Na_2_CO_3_ (20%) and 1.9 mL of distilled water were added. Then, the mixture was incubated in darkness for 2 h. The absorbance of the solution was measured at 760 nm. A standard curve was performed using gallic acid, and the results are expressed as the µg equivalent of gallic acid/mg dw.

### Antioxidant activity (ABTS assay) of plant shoot and seed extracts

Using the method outlined in Stratil et al. (2006); Stratil et al. (2006), the antioxidant activity of both plant and seed extracts against ABTS was assessed. Through the oxidation of ABTS by potassium persulfate, radical ABTS^·+^ was created. A mixture of potassium persulfate (4.95 mM) and ABTS (7 mM) in a 1:1 ratio was combined and stored in darkness for 16 h at 24°C. Methanol was used to dilute the mixture until its absorbance value was in the range of (1–1.1) at 734 nm. Then, 3.9 mL of the diluted ABTS^·+^ mix was added to a 0.1 mL methanolic extract of each sample. Triplicate measurements were performed for plant and seed extract samples. Ascorbic acid was used for the standard curve, and the results are expressed as the µg equivalent of ascorbic acid/mg dw.

### Biosynthesis of bimetallic Ag@Se-P and Ag@Se-S nanoparticles

Ag@Se-P nanoparticles were synthesized using the *P. aculeata* leaf (PAL) extract in a 1:1 M ratio of Ag:Se according to the method described in Hassanin et al. (2021). In detail, 25 mL of a 10 mM AgNO_3_ solution was mixed with 25 mL of a 10 mM Na_2_SeO_3_ solution, followed by the addition of 25 mL of the plant extract while stirring at room temperature for 24 h. Then, the produced material was collected by centrifugation at 6,000 rpm, washed several times using deionized water, and then dried. This method was repeated to prepare Ag@Se-S nanoparticles that were synthesized using the *P. aculeata* seed (PAS) extract.

### Characterization

The FT-IR spectrophotometer, model number 360, from Cary, South San Francisco, United States, was used to measure FT-IR of all samples. The optical properties of the synthesized materials were examined using a UV-Vis spectrophotometer (Shimadzu, Kyoto, Japan). A FEI Quanta 250 FEG high-resolution field emission scanning electron microscope, fitted with a high-angle, angular dark-field detector, and an x-ray energy-dispersive spectroscopy (EDS) system was used to detect the morphology, size, and crystallinity of the particles. Transmission electron microscope model JEOL JEM-2100, Tokyo, Japan, was applied to collect the TEM images of the synthesized materials at an acceleration voltage of 90 KV. X-ray powder diffraction (XRD) was used to identify the crystalline phase of nanoparticles. An X-ray diffractometer (EMPYREAN by Cu Ka radiation with a wavelength of 1.54◦A) was used to identify the crystalline phases.

### Antioxidant activity (DPPH) of bimetallic nanoparticles

The nanoparticles’ antioxidant activity was assayed following the method of Katalinic et al., 2006; Katalinic et al., 2006). The blend of 1 mL of each plant and seed nanoparticle with 2 mL of methanolic DPPH (10^−4^ M) was kept in the dark at room temperature for 16 min. The absorbance of the samples was measured at 517 nm. Methanol was used instead of the nanoparticle solution as a blank. Gallic acid was used for the standard curve, and the data are expressed as the mg equivalent of gallic acid/g dw.

### Cytotoxicity assay

The MTT test was used to assess the cytotoxicity of Ag@Se-S and Ag@Se-P against fibroblast cells. L929 fibroblast cells were seeded on a 24-well cell culture plate and cultured for 24 h at 37 °C in a 5% CO_2_ incubator. Following the incubation time, the cells were treated with various doses of Ag@Se-S and Ag@Se-P (control, 2.5, 5, 7.5, 10, and 15 Mg/mL) and incubated for 24 h in a 5% CO_2_ incubator at 37°C. The cells were then washed with PBS and treated for 4 h in the dark with serum-free media containing 10 µL MTT (0.5/mL). The generated insoluble formazan crystals were dissolved in 100 µL of DMSO solution, and the absorbance of each sample was measured at 545 nm using a microplate reader.

### Scratch wound assay

Ag@Se-S and Ag@Se-P wound healing properties were investigated *in vitro* utilizing a wound scratch experiment previously reported by Liang et al. (2007); Liang et al. (2007). L929 fibroblast cells were seeded onto a 6-well culture plate and incubated until 90% confluency was achieved. When the plates reached 90% confluence, they were scraped vertically with a sterile micropipette. The width of the scratch was kept consistent throughout all wells. The scratched cells were washed with fresh DMEM before being treated with 5 and 7.5 g of Ag@Se-S and Ag@Se-P, respectively. The cells were then cultured for 24 h at 37°C in a 5% CO_2_ incubator. Following the incubation time, the wound closure was examined microscopically, and photographs were taken. The cells’ relative migration ratio (RMR) was calculated using Eq. 1.

$$RMR=\left(\frac{A_{0}-A_{1}}{A_{0}}\right)x\;100,$$ (1)

where A_0_ is the area of scratch made initially and A_1_ is the area of scratch after 24 h of incubation.

### Enzyme-linked immunosorbent assay

IL-1, IL-6, and TNF-alpha levels were determined using an enzyme-linked immunosorbent assay (ELISA) kit (R&D Systems, Minneapolis, MN) according to the manufacturer’s instructions. At 450 nm, the absorbance was measured, and the subtraction was measured at 540 nm. The concentration of inflammatory markers in each well was then determined using its corresponding standard curves.

### Reverse transcription-polymerase chain reaction

Methods and conditions were as previously published (Ahmed et al., 2022). LPS (1 μg/mL) was applied to RAW 264.7 cells in the presence or absence of Ag@Se-S and Ag@Se-P. Total RNA was obtained by harvesting whole-cell lysate using triazole LS reagents (Invitrogen, Carlsbad, CA) according to the manufacturer’s procedure. Thermo Fisher Scientific cDNA was employed to synthesize 1st stranded cDNA, utilizing total RNA (2 g)

### Protein marker quantification using Western blot

Immunoblotting was performed, as previously explained by Ibrahim et al. (2022). According to the supplied methodology, proteins were extracted from cells at ice-cold conditions using the PRO-PREP^TM^ Protein Extraction Solution from iNtRON Biotechnology in Seongnam-Si, Korea (Seongnam, Gyeonggi-Do, South Korea). SDS-polyacrylamide gel electrophoresis (PAGE) at 8% was used to separate 30 μg of total protein per lane. After that, proteins were transferred to nitrocellulose membranes (Millipore), which were then blocked with 5% skimmed milk and probed with the appropriate primary antibodies after 1.5 h of incubation. The membranes were washed five times (5 min per wash) and then incubated for 2 h with horseradish peroxidase-conjugated secondary antibodies. The blot was then detected using an enhanced chemiluminescence system (Amersham Biosciences Inc., Piscataway, NJ) and exposed to X-ray film (Fuji Photo Film Co., Ltd.).

## Results and discussion

### Characterization of synthesized nanoparticles

#### FT-IR spectroscopy

**FIGURE 1:**
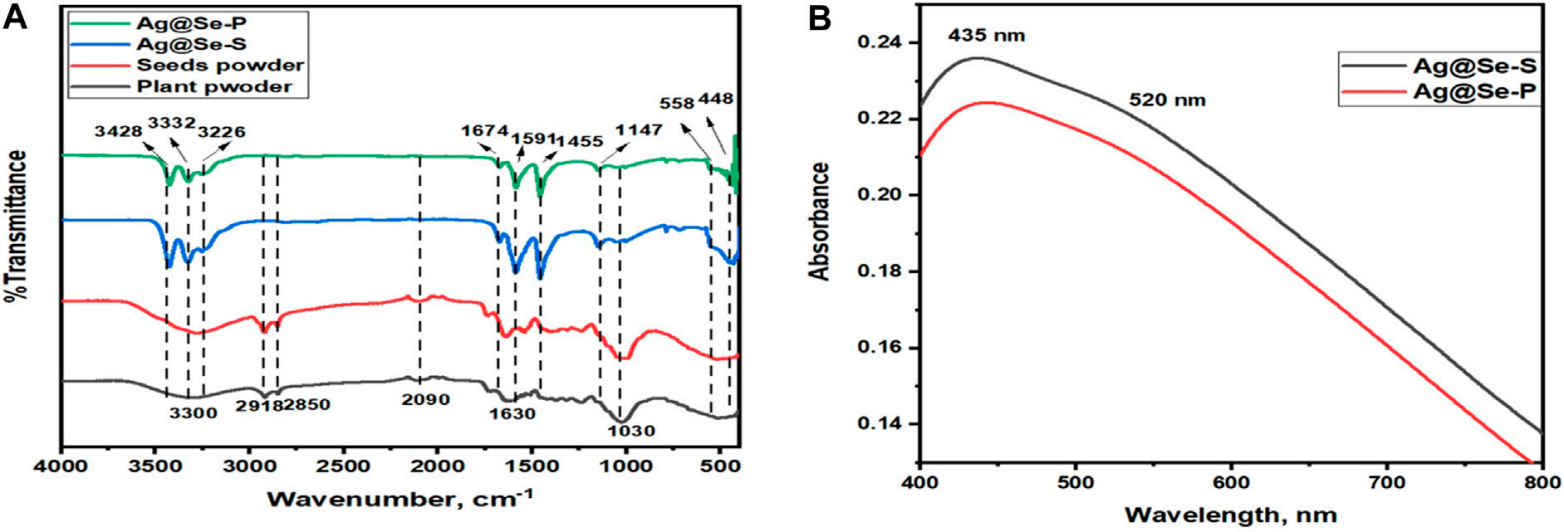
**(A)** FT-IR spectra for Ag@Se-P, Ag@Se-S, and *Parkinsonia aculeata L.* seed and plant powders **(B)** UV-visible spectra for Ag@Se-P and Ag@Se-S nanocomposites.

FT-IR analysis was used to identify phytochemicals in the plant and seed extracts responsible for nanoparticle production, capping, and stabilization. The FT-IR spectrum for both plant and seed powders is identical, indicating that both parts of the plants have almost the same phytochemical structure, as shown in Figure 1A. The broadband between 3,600 and 3,000 cm^−1^ and centered at ≈ 3,300 cm^−1^ could be assigned to the hydrogen-bonded N-H/O-H group of amines, amides/alcoholic compounds, and phenolic compounds (Wei et al., 2015; Mabasa et al., 2021). The small bands at 2,918 and 2,850 cm^-1^ are attributed to C-H stretching vibrations for CH_2_ and CH_3_ groups, indicating the presence of terpenes (Khoza et al., 2016; Mabasa et al., 2021). The stretching vibrational modes of C≡C and C=C groups of alkynes and alkenes appear at the absorption bands 2090 and 1,630 cm^−1^, respectively (Alara et al., 2018). The band at 1,030 cm^−1^ is assigned to the C-O stretching vibration of secondary alcohols or ester groups (Syed Ali Fatima. and Johnson, 2018). The FT-IR spectra of the synthesized materials Ag@Se-P and Ag@Se-S show major peaks at 3,428, 3,332, 3,226, 1,674, 1,591, 1,455, and 1,147 cm^−1^. These peaks matched the phytochemicals attached to the surface of the produced nanomaterials. The absorption bands at 558 and 448 cm^−1^ correspond to Se-O and Ag-O vibrational modes, respectively (Nandini et al., 2017; Hassanin et al., 2021). These results demonstrate that the biomolecules of *P. aculeata L.* have a potential effect on the production of Ag@Se-P and Ag@Se-S nanomaterials.

#### UV-visible analysis

The optical properties of the synthesized materials were examined using UV-vis spectroscopy to confirm the successful synthesis of the prepared materials. Figure 1B represents the UV-visible spectra for Ag@Se-P and Ag@Se-S nanomaterials. As shown in the figure, the surface plasmon resonance of both samples consists of two main peaks occurring at 435 and 520 nm, which correspond to Ag and Se nanoparticles, respectively. These data are consistent with those found in the available literature values (Ahmad et al., 2020; Ahmed et al., 2020; Hassanin et al., 2021), revealing the effective synthesis of Ag@Se nanocomposites.

#### SEM analysis

**FIGURE 2:**
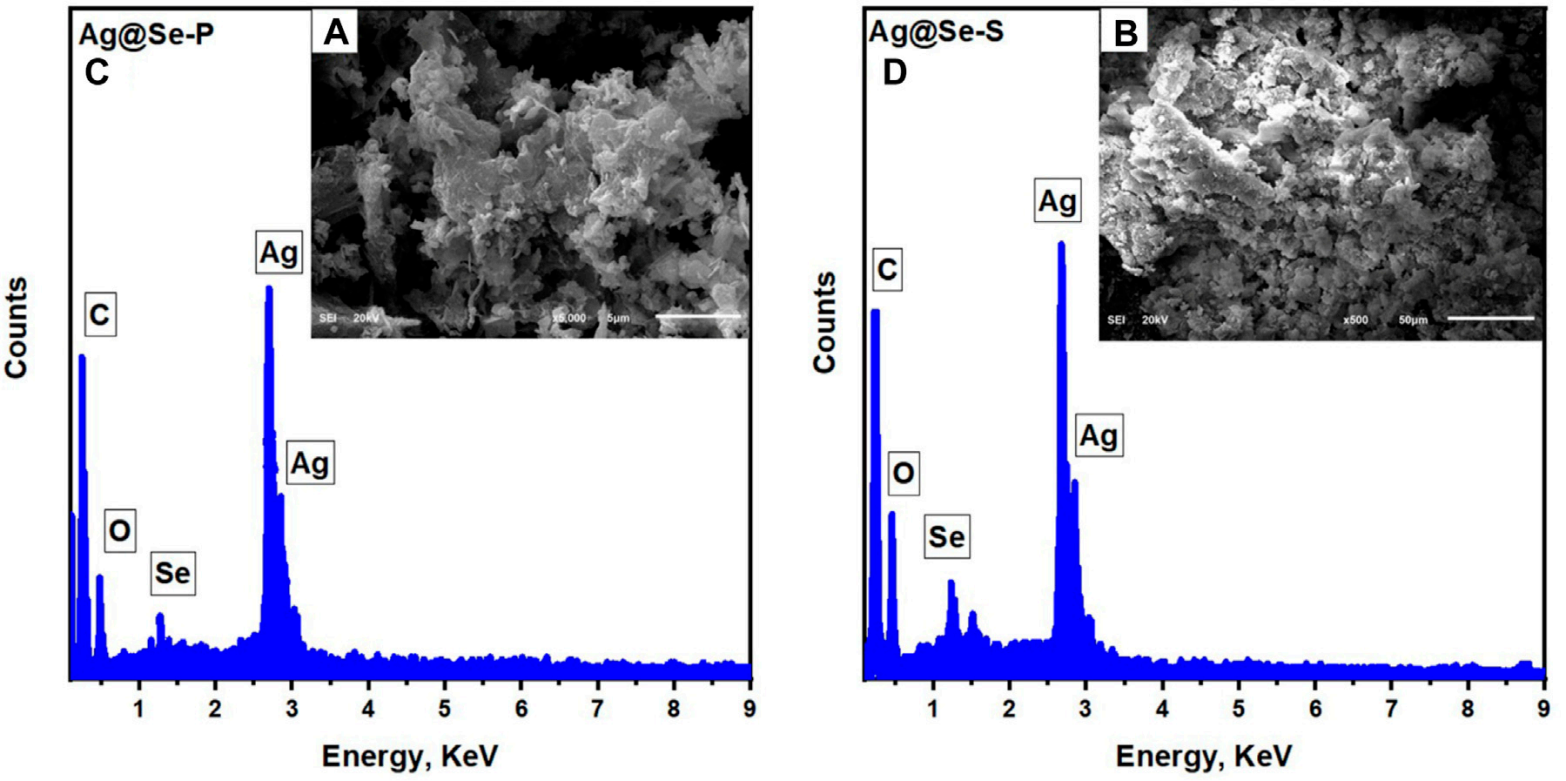
EDS analysis with the inset SEM image for the synthesized **(A)** Ag@Se-P nanocomposite using the park extract and **(B)** Ag@Se-S using seeds extract.

The morphological structure and elemental constituents of the synthesized nanocomposites were examined using scanning electron microscopy (SEM) and EDS. Figures 2A, B show SEM images for the synthesized Ag@Se-P and Ag@Se-S. It can be observed from the images that the synthesized Ag@Se-P and Ag@Se-S have clustered irregular spherical shapes (Maheshwari and Verma, 2017; Hassanin et al., 2021). The presence of biomolecules from the *P. aculeata L.* aqueous extract attached to the surface of the synthesized materials is attributed to the agglomeration and cluster formation of Ag@Se-P and Ag@Se-S nanocomposites, which confirm the role of the *P. aculeata L*. aqueous extract as a reducing and capping agent (Hassanin and Taha, 2022; Numan et al., 2022). Figures 2C, D show the elemental mapping obtained from EDS analysis for the produced Ag@Se-P and Ag@Se-S nanocomposites, respectively. It can be seen from the figure that the formed nanocomposites consist of Ag, Se, C, and O. The coexistence of carbon and oxygen could be attributed to the biomolecules linked to the nanocomposites in the plant extract (Ahmad et al., 2020).

#### XRD analysis

**FIGURE 3:**
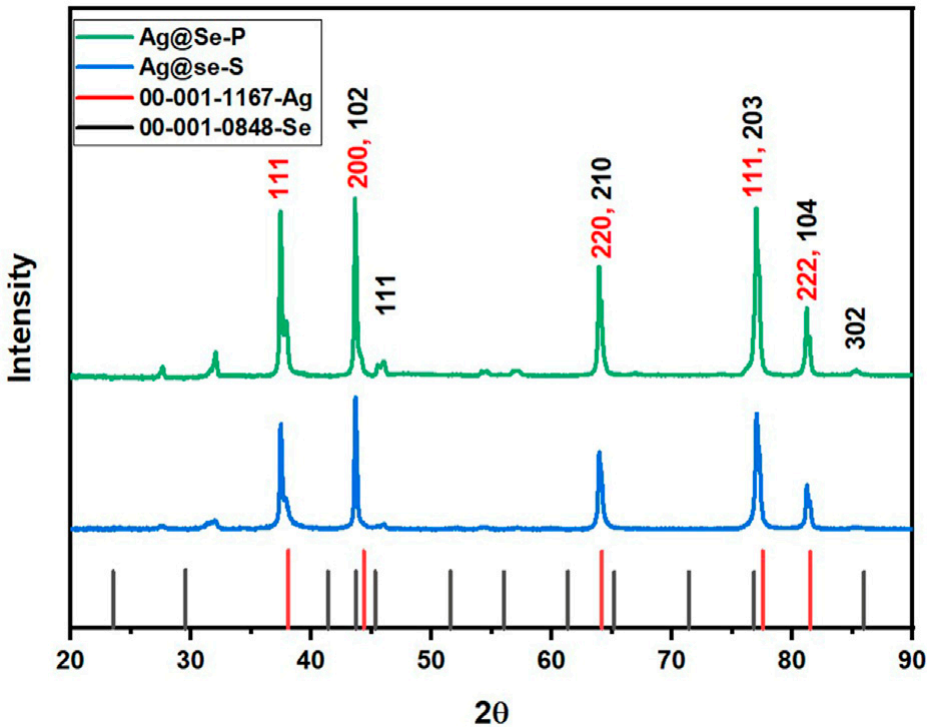
X-ray diffraction patterns of the synthesized Ag@Se-P and Ag@Se-S nanocomposites.

XRD was used to examine the crystal structure of the prepared materials. Figure 3 shows the XRD pattern of Ag@Se-P and Ag@Se-S nanocomposites. It can be seen from the figure that both samples have almost identical peaks. The peaks at 2θ values of 38.1, 44.3, 64.1, 77.5, and 81.5 correspond to the diffraction plans 111, 200, 220, 311, and 222, respectively. These diffraction plans are characteristic of the cubic crystalline phase of silver with a space group (Fm-3m) according to JCPDS card no. 00-001-1,167 (Taheri et al., 2022). However, the peaks at 43.7, 45.3, 65.1, 76.8, 81.5, and 85.9 are attributed to the hexagonal crystallite system of selenium (Se) that match the diffraction plans 102, 111, 210, 203, 104, and 302 (JCPDS #00-001-0848), respectively (Morad et al., 2022). The crystallite size for the manufactured materials was calculated using the Debye–Scherrer equation (Eq. 2) (Kroon and Ted, 2013).

$$D=\frac{K\lambda }{\beta \;\cos\theta },$$ (2)

where K refers to the Scherrer constant, λ is the X-ray beam wavelength (1.54,184 Å), β represents the full width at half maximum (FWHM) of the peak, and θ is the Bragg angle.

The average particle size of the synthesized materials was calculated and found to be 17.65 and 24.36 nm for Ag@Se-S and Ag@Se-P, respectively.

#### HR-TEM analysis

**FIGURE 4:**
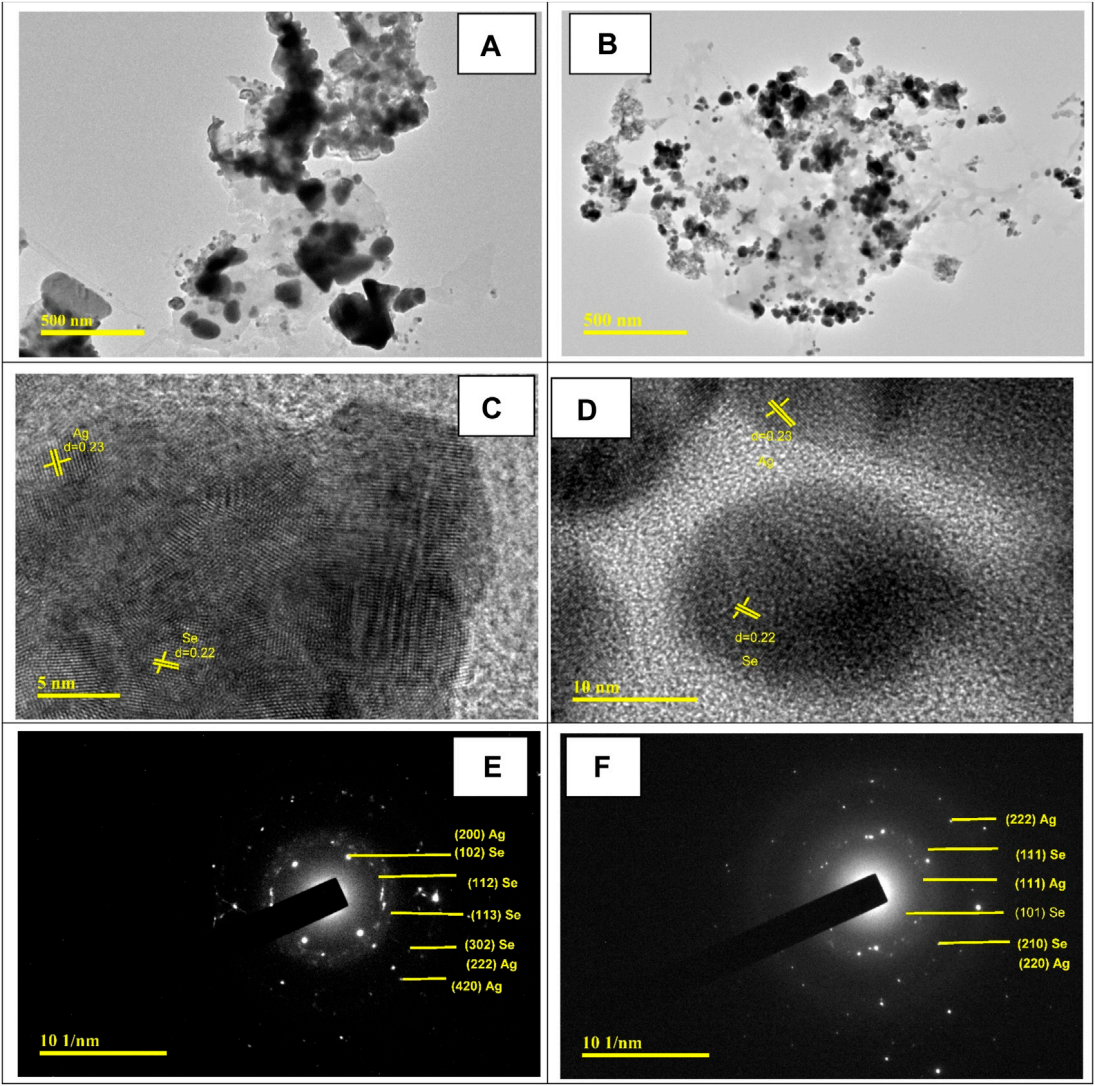
HR-TEM images for **(A)** Ag@Se-P and **(B)** Ag@Se-S. Magnified TEM images with calculated lattice fringes for **(C)** Ag@Se-P and **(D)** Ag@Se-S and SAED patterns for **(E)** Ag@Se-P and **(F)** Ag@Se-S.

Figure 4 shows the HR-TEM images of the prepared Ag@Se-P and Ag@Se-S nanocomposites. Both nanocomposites are agglomerated with irregular spherical shapes, as shown from TEM images in Figures 4A, B. Figures 4C, D show the magnified images of Ag@Se-P and Ag@Se-S nanocomposites with the calculated lattice fringes of 0.23 nm (111) and 0.22 nm (110) related to the d-spacing of Ag and Se, respectively (JCPDS card no. 00-001-1167 and 00-001-0848) (Chen and Penn, 2019; Ahmad et al., 2020). These results confirm the successive formation of the Ag@Se nanocomposite. Selected area electron diffraction (SAED) analysis was used to further confirm the synthesis of the Ag@Se nanocomposite. SAED patterns of Ag@Se-P and Ag@Se-S nanocomposites in Figures 4E, F illustrate polycrystalline diffraction rings that are aligned with the reflections of Se (111, 101, 210, 102, 112, 113, and 302) and Ag (111, 220, 200, 222, and 420), which are in good agreement with the XRD results (Enshirah et al., 2018; Ahmad et al., 2020).

**FIGURE 5:**
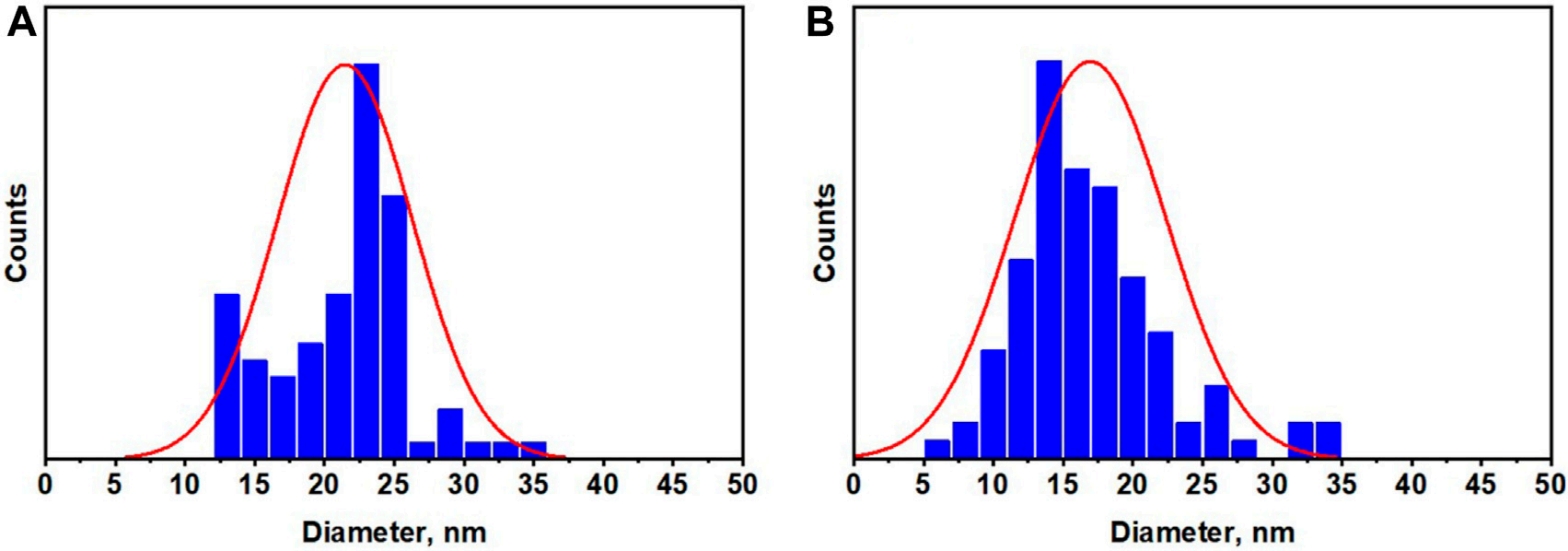
Particle size distribution for the synthesized **(A)** Ag@Se-P and **(B)** Ag@Se-S nanocomposites.

The particle size distribution was calculated from TEM measurements using ImageJ software and presented in Figures 5A, B. The calculated average particle size was found to be 21.4 and 16.95 nm for Ag@Se-P and Ag@Se-S, respectively, which are in good agreement with XRD results, indicating the successive synthesis of Ag@Se-NC using the *P. aculeata* L. aqueous extract.

### Biological activity of synthesized nanoparticles

#### Total phenol estimation of plant shoot and seed extracts

The measured values of total phenols in both plant leaves and seed extracts are presented in Table 1. The leaf extract of *P. aculeata* had almost as much as double the concentration of total phenol in the seed extract with values of 37.2 and 19.2 mg equivalent gallic acid/g dw for leaves and seeds, respectively. Due to their hydroxyl groups’ capacity to scavenge free radicals, phenols are crucial secondary metabolites in plants that increase the antioxidant activity of the plants (Pratyusha, 2022).

**TABLE 1:** Total phenol and antioxidant activity of leaf and seed extracts as well as leaf and seed nanoparticles of *P. aculeata*.

Extract (0.1 mg/mL)	Total phenol (mg GAE/g dw)	Nanoparticle (mg GAE/g dw)	Antioxidant (µg ASE/µL)
Leaves	37.2^a^ ± 1.4	57.2^a^ ± 1.8	8.7^a^ ± 0.4
Seeds	19.2^b^ ± 1.8	43^b^ ± 1.9	7.7^b^ ± 0.8

*Each value in the table is the mean of three replicates ± SD. Different letters in each column indicate significant difference at *p* < 0.05. GAE, gallic acid equivalents; ASE, ascorbic acid equivalents; dw, dry weight.

#### Antioxidant activity of plant leaf and seed extracts

**FIGURE 6:**
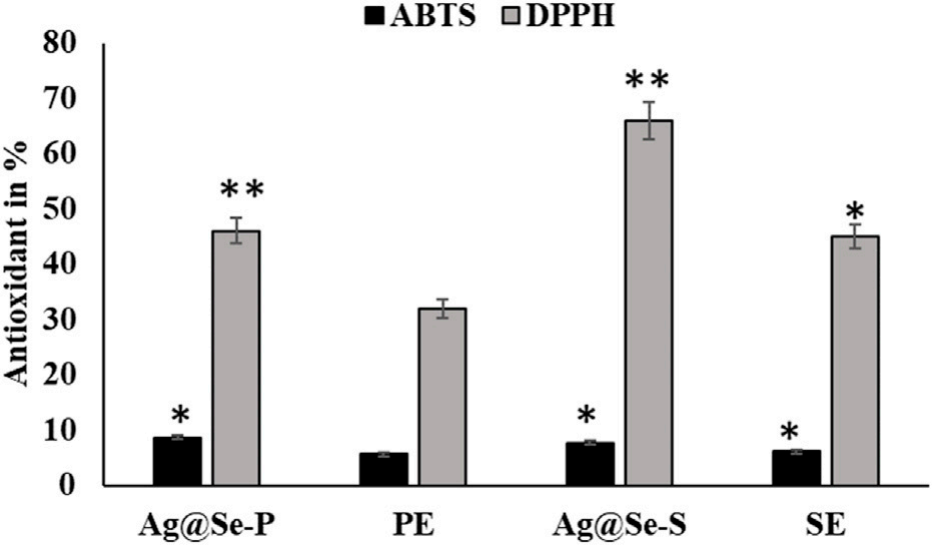
Antioxidant activity of seed and plant extracts with silver selenite nanoparticles. DPPH and ABTS methods were used for seed and plant extracts with nanoparticle silver selenite.

Medicinal plants are nature’s gift for humans and are extensively used for healthcare and the cure of diseases (Mohammed et al., 2020). Seeking new plant-based antioxidants is crucial for therapeutic strategies for different illnesses (Abdelaziz et al., 2020). The values of the antioxidant activity for the *P. aculeata* leaf and seed extracts measured by the ABTS assay are displayed in Table 1; Figure 6. Regarding the antioxidant activity, the leaf extract had a higher antioxidant value (8.7 µg ASE/µL compared to the seed extract (7.7 µg ASE/µL).

#### Antioxidant activity (DPPH)

The antioxidant activity of the biosynthesized bimetallic nanoparticles Ag@Se-P and Ag@Se-S was evaluated using the DPPH assay (Table 1; Figure 6). The color of the DPPH solution containing the biosynthesized bimetallic Ag@Se nanoparticles from the *P. aculeate* leaf extract changed, indicating their ability to scavenge free radicals. The antioxidant potential of the synthesized nanoparticles is indicated by the color change from violet to light yellow, which was further measured using a spectrophotometer. At the same concentrations of seed and plant extracts (0.1 mg/mL), the Ag@Se-S nanoparticles showed higher inhibition than Ag@Se-P (37.2 and 19.2, respectively). In nanoparticles, the results showed 57.2 and 43 activity equivalents to gallic acid. These results were compared with standard ascorbic acid. As demonstrated in Table 1, Ag@Se-P nanoparticles are more potent antioxidants than ascorbic acid because their half-maximal inhibitory concentration (IC_50_) values for Ag@Se-P and ascorbic acid are 33.85 g/mL and 50 g/mL, respectively. Ag-NPs created from *Elephantopus scaber* leaf extract have been shown to exhibit higher DPPH scavenging activity, according to similar observations (Kharat and Mendhulkar, 2016). The results of this investigation show that the synthesized Ag@Se nanoparticles have antioxidant properties in terms of their capacity to scavenge free radicals. Using the same process, ascorbic acid was used as a reference standard, and the DPPH free radical assay was used to evaluate the antioxidant activity of the *Hagenia abyssinica* plant leaf extract. The *H. abyssinica* plant leaf extract has less antioxidant activity in terms of scavenging free radicals than Ag-NPs (Melkamu and Bitew, 2021).

#### Cytotoxic activity

**FIGURE 7:**
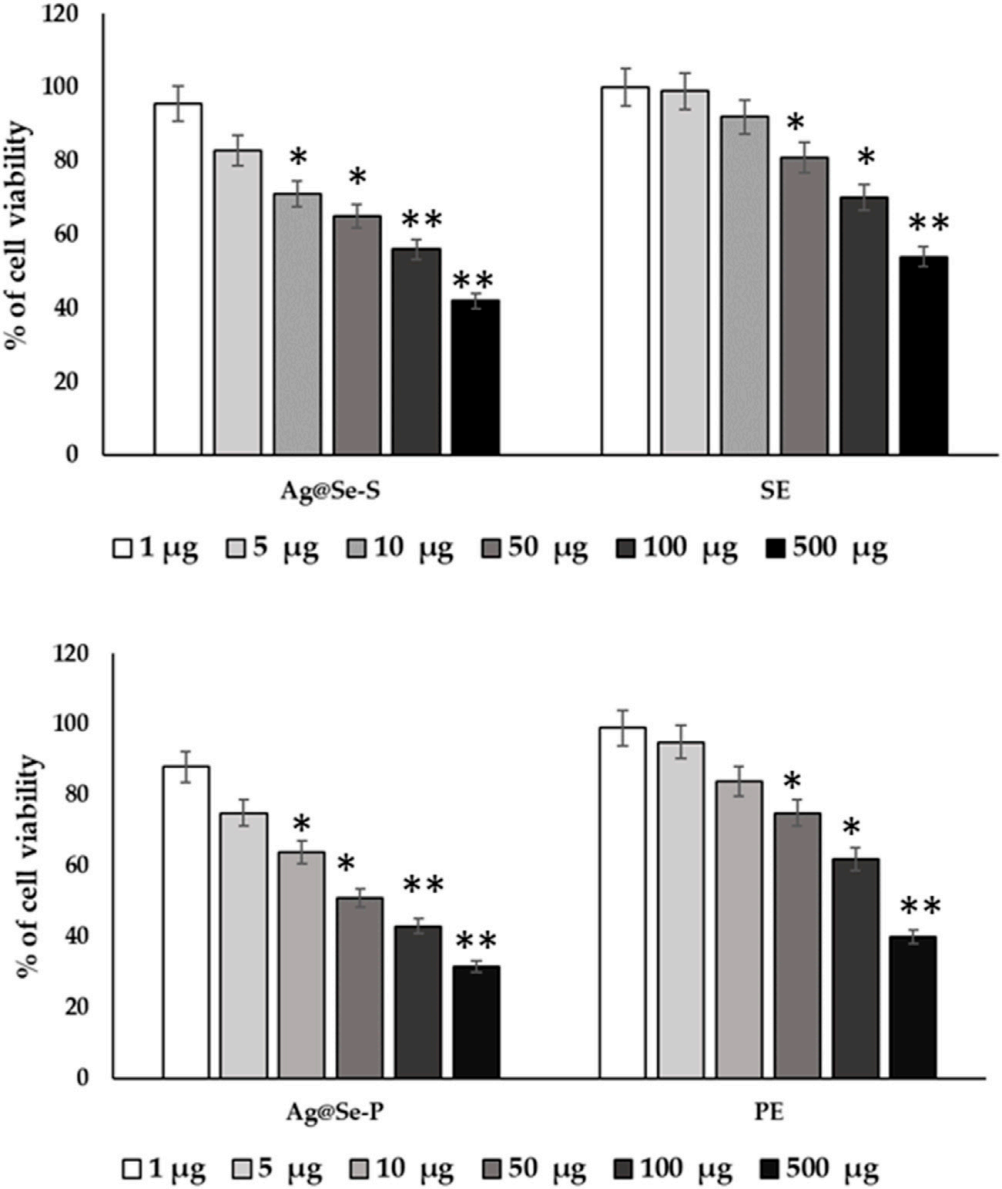
*P. aculeata* seed and plant extract alone and with nanoparticle coupled with seed and plant extracts were evaluated for the proliferation rate of murine macrophage cell lines (RAW 264.7).

RAW 264.7 cell lines used in cytotoxic tests show toxic effects on cell survival. The percentage of cell viability declines as concentration increases (Figure 2). Natural plant extracts of *P. aculeate* are used in the green synthesis of nanoparticles. In this study, the *in vitro* RAW 264.7 cell line was used as a model to investigate the inhibitory effect of Ag@Se-S and Ag@Se-P nanoparticles and seed and plant extracts with and without nanoparticles for toxic dose determination. The aim of this study was to investigate the safety of nanoparticles and reduce cell toxicity using the MTT assay. The ability of RAW 264.7 cells to survive Ag@Se-P nanoparticles, as seen in Figure 7, displayed no harmful effects at concentrations ranging from 1 to 25 g/mL. Additionally, Ag@Se-S is less toxic than Ag@Se-P. The leaf extract alone is more toxic than the seed extract. The concentration up to 25 ug/mL was not toxic against RAW 264.7 cells. Therefore, 10 ug/mL or lower doses were used in the tests that came after.

Cells were supplemented with Ag@Se-S and Ag@Se-P nanoparticles and seed and plant extracts (0–100 μg) for 48 h time periods, and cell viability was assessed using MTT assays. Cells were treated with LPS (10 ng/mL) or a vehicle control (0.1% DMSO). Cell viability was quantified after incubating cells with atropine for 48 h for normal cells (A). The seed extract was supplemented with RAW 264.7 cells, and the cell viability was evaluated after a time point of 48 h. (B) The plant extract was supplemented with RAW 264.7 cells, and the cell viability was evaluated after a time point of 48 h. The results are expressed as the mean ± SD of triplicate measurements.

#### Anti-inflammatory activity

**FIGURE 8:**
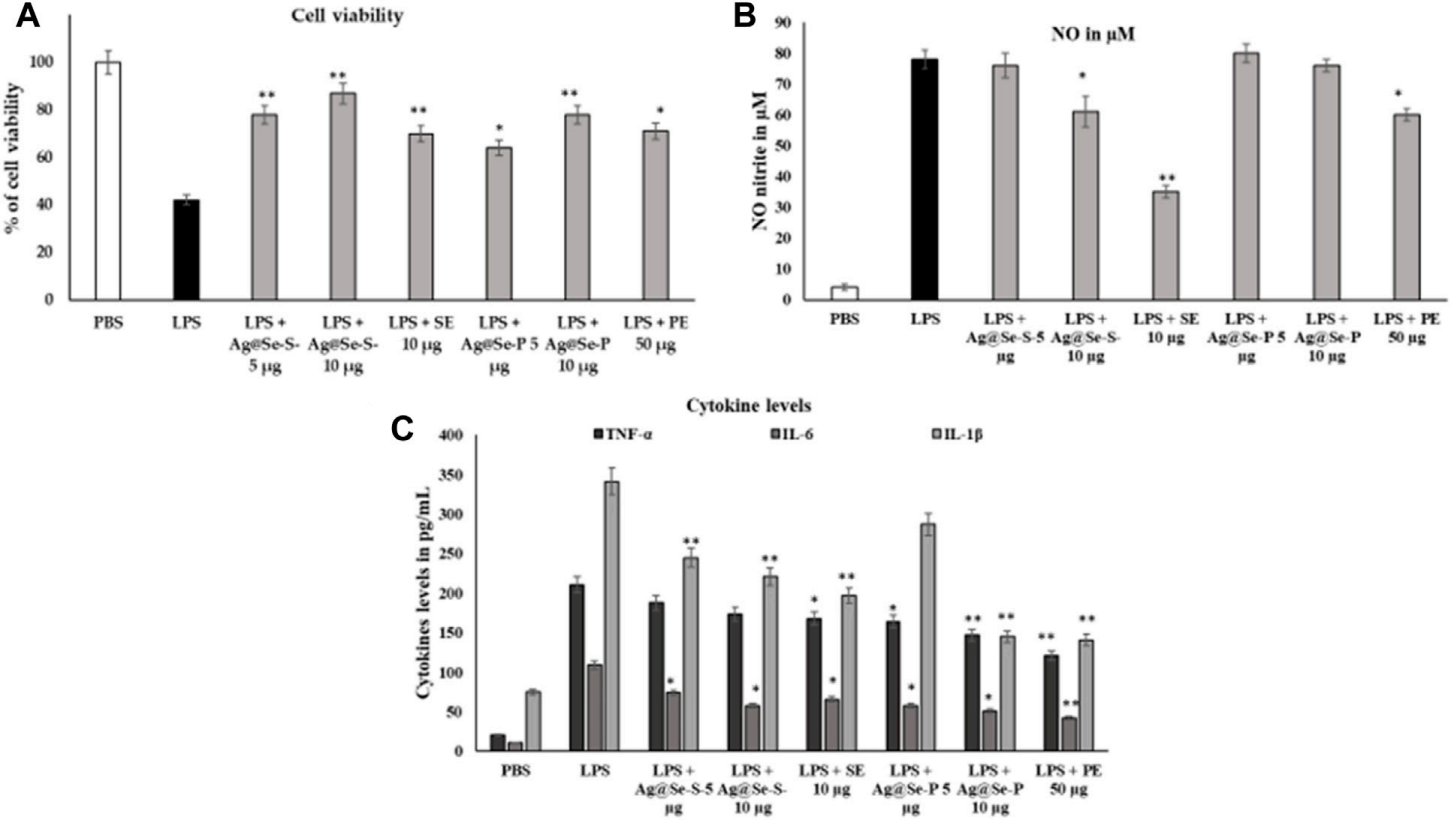
Effects of Ag@Se-S and Ag@Se-P nanoparticles and seed and plant extracts on cell viability and morphology of RAW 264.7 cells. RAW 264.7 macrophages were cultured with PA seed extract and plant extract for 24 h, and then cell viability based on MTT assay (Figure 8A). LPS induced cells supplemented with PA seed extract and plant extract. Effects of PA seed extract and plant on the production of nitric oxide (NO), reactive oxygen species (ROS), and tumor necrosis factor-alpha (TNF-α) in lipopolysaccharide (LPS)-stimulated RAW 264.7 cells. Cells were pretreated with PA seed extract and plant extract for 24 h. After washing with phosphate-buffered saline (PBS), cells were stimulated with 1 μg/mL LPS for 18 h. the cell viability RAW 264.7 on PA seed extract and plant extract treatment after 48 h **(A)**. The level of NO in the medium was quantified by Griess reagent **(B)**. The levels of TNF-α, IL-6 and IL-1B in RAW 264.7 cells were analyzed by an ELISA **(C)**. Values represent the mean ± SD (*n* = 3). Statistical significance was calculated by t-test and one-way ANOVA. ^#^ *p* < 0.05 vs. cells treated with media only; n. s., not significant; ** *p* < 0.001 vs. cells treated with LPS only.

LPS causes macrophages to engage in inflammatory processes. In the LPS-challenged model, 10 ug/mL of Ag@Se-S and Ag@Se-P nanoparticles recover the viability and inhibit the LPS-induced toxicity in RAW 264.7 cell lines (Figure 8).

Pandiyan et al. (2022) found that Se-NPs synthesized using *Thymus vulgaris*, a medicinal plant, exhibited antioxidant and anti-inflammatory properties. Moreover, crude or whole plant extracts have been reported to have superior activity over isolated single constituents through the additive and/or synergistic effects of bioactive compounds (Long et al., 2015). This supports the idea of using crude extract for enhancing the biological activity of the nanoparticle. In this study, we found that both Ag@Se-S and Ag@Se-P nanoparticles and seed and plant extracts showed higher anti-inflammation activity than Ag@Se-NPs by decreasing the nitric oxide level through the reduction of cytokine protein in inflamed cells.

These cytokines are crucially implicated in inflammation, various auto-immune illnesses, and the development of tumors, according to numerous studies. We used the nitrite oxide and cytokine TNF-α, IL-6, and IL-1β estimation, which is a result of NO and cytokines, to assess the inhibitory activity of Ag@Se-S and Ag@Se-P inflammatory mediators, which significantly blocked LPS-induced NO and inflammatory cytokines. When compared to the LPS-induced control group, nitrite analysis showed that the treatment of both samples might limit nitrite activity. LPS significantly activated the cytokines, and seed and plant extracts with and without NPs were actively suppressed by the cytokine expression and release in RAW 264.76 cell lines (Figure 8C). The anti-inflammatory effects of extracts from bryophyte species on NO production inhibition were the subject of other studies. As a result, LPS-stimulated RAW 264.7 cells (500 ng/mL, 24 h) were treated with 50 g/mL of the peat moss (Sphagnum sp.) aqueous extract to decrease the formation of NO (Choi et al., 2014). The NO generation caused by the treatment of LPS (1 g/mL, for 24 h) was found to be inhibited by the *P. aculeate* nanoparticles with an IC_50_ value of 10 μg/mL. In contrast, some previous studies showed the anti-inflammatory properties of the *Polytrichastrum formosum* (Bryophyta) extract detected throughout our screening. Plant species may differ in their ability to reduce inflammation due to differing chemical components. This can also change with the geographic origin of the species and exposure to various environmental factors (season, soil, climate, etc.) (Peters et al., 2018; Commisso et al., 2021).

As previously reported, the macrophage-like RAW 264.7 cells describe the action of numerous anti-inflammatory mechanisms at the molecular stage; therefore, further investigation was conducted to explore whether gold and silver nanoparticles can function as inhibitors of nitric oxide production (Ahn et al., 2016).

RAW 264.7 macrophages were cultured with PA seed and plant extracts for 24 h, and then cell viability was carried out based on the MTT assay (A). LPS-induced cells were supplemented with PA seed and plant extracts. Effects of PA seed and plant extracts on the production of nitric oxide (NO), reactive oxygen species (ROS), and tumor necrosis factor-alpha (TNF-α) in lipopolysaccharide (LPS)-stimulated RAW 264.7 cells were assessed. The cells were pretreated with PA seed and plant extracts for 24 h. After washing with PBS, the cells were stimulated with 1 μg/mL LPS for 18 h. The cell viability RAW 264.7 on PA seed and plant extract treatment after 48 h (A). The level of NO in the medium was quantified by Griess reagent (B). The levels of TNF-α, IL-6, and IL-1B in RAW 264.7 cells were analyzed using the ELISA (C). Values are represented as the mean ± SD (*n* = 3). Statistical significance was calculated using the *t*-test and one-way ANOVA. #*p* < 0.05 vs. cells treated with media only; n.s., not significant; ***p < 0.001 and ****p < 0.0001 vs. cells treated with LPS only.

#### Scratch wound healing capacity

**FIGURE 9:**
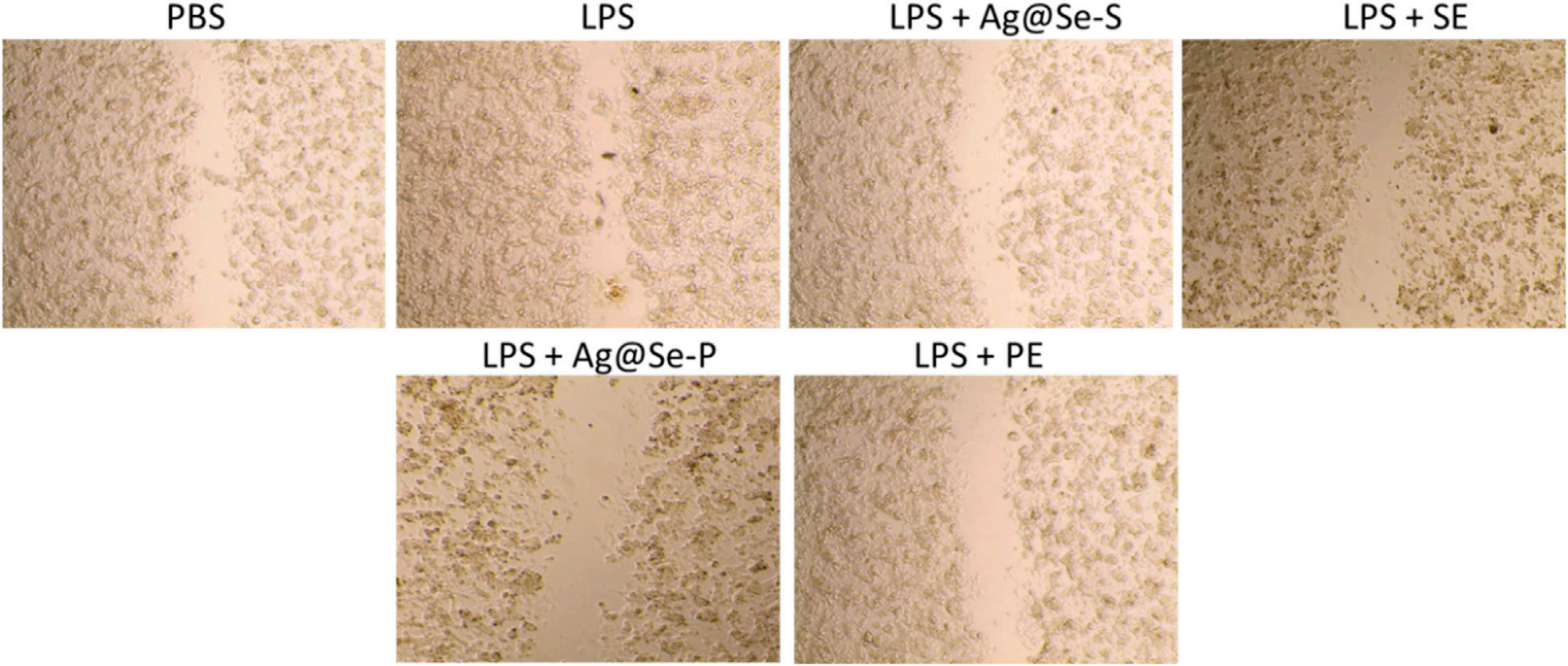
Effects of PA seed and plant extracts on the cell migration of RAW 264.7 cells using the scratch wound assay. Magnification of the region framed ×20 objective lens with magnification power ×200.

The wound scratch assay was used to evaluate the wound healing capacities of Ag@Se-S and Ag@Se-P wound healing capacities, and the results are shown in Figure 9. RAW 264.7 cells’ migration was inhibited by treatments with Ag@Se-S and PAS extract alone, Ag@Se-P, and PAA extract alone in 10 μg/mL. The migration of RAW macrophage cells was dramatically reduced after treatment with 10 μg/mL of Ag@Se-S and Ag@Se-P.

Our results align with the literature that claims Ag@Se-S and Ag@Se-P nanoparticles and seed and plant extracts facilitate wound healing by promoting fibroblast differentiation in myofibroblasts, wound contractility, and epidermal re-epithelialization via keratinocyte migration and proliferation (Vijayakumar et al., 2019). Similar findings where plant-derived Ag-NPs improved wound healing through fibroblast and keratinocyte migration have previously been described (Ahn et al., 2019; Annamalai et al., 2019).

**FIGURE 10:**
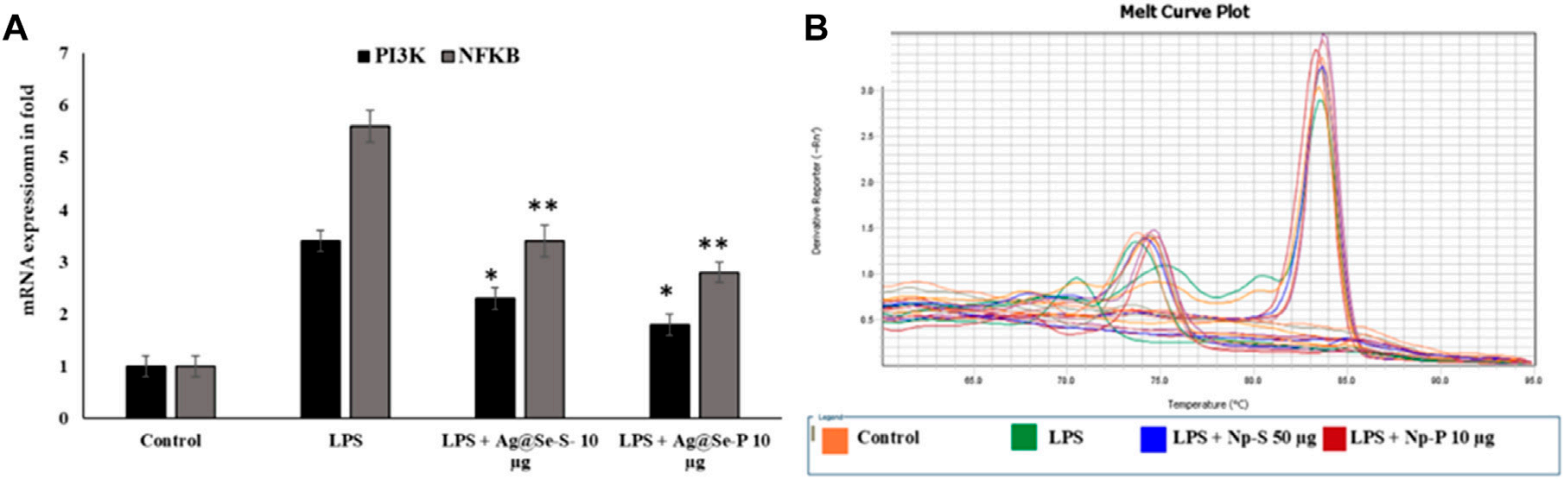
Effects of Ag@Se-S and Ag@Se-P nanoparticles and seed and plant extracts on the mRNA expression of pro-inflammatory markers in LPS-stimulated RAW 264.7 macrophages. **(A)** the mRNA expression was noted in fold and actin used as internal control. **(B)** melting curve of specific genes used in RT-PCR. Statistical significance was calculated using the t-test and one-way ANOVA. ^#^p < 0.05 vs. cells treated with media only; n.s., not significant; *p < 0.05 and **p < 0.01 vs. cells treated with LPS only.

**FIGURE 11:**
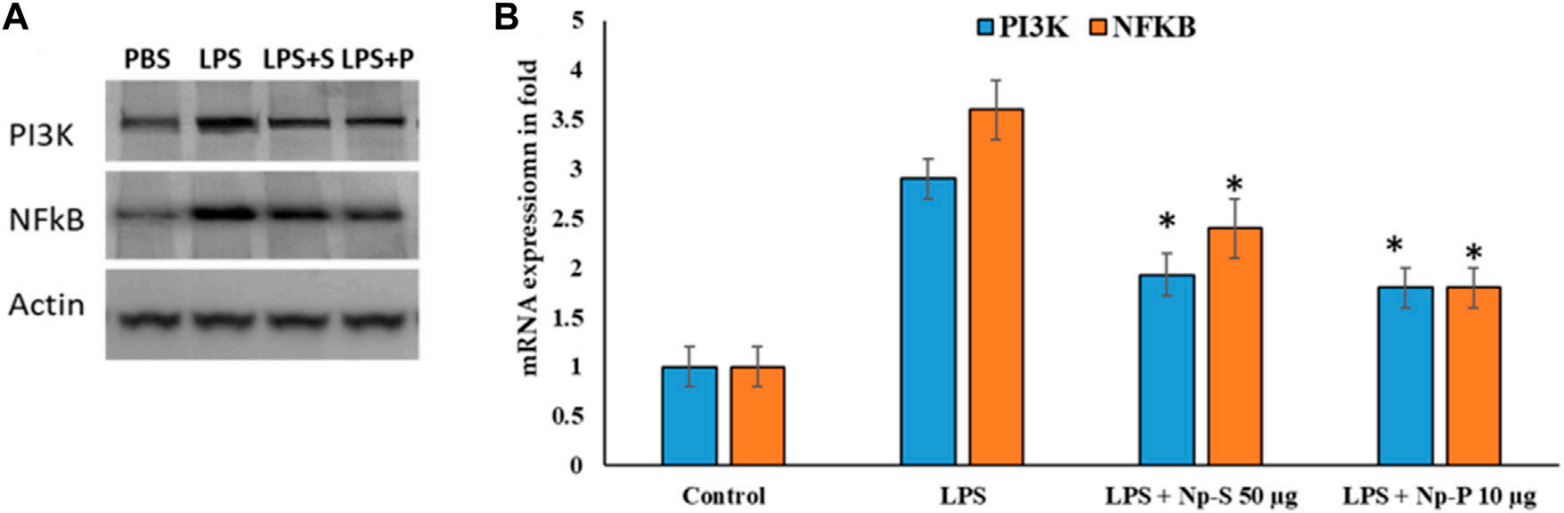
Effects of Ag@Se-S and Ag@Se-P nanoparticles and seed and plant extracts on LPS-induced activation of the PI3K and NF-κB pathway in RAW 264.7 cells. **(A)** Protein western blot of PI3K and NF-κB and Actin used as internal control and **(B)** expression values in fold. Statistical significance was calculated using the t-test and one-way ANOVA. #*p* < 0.05 vs. cells treated with media only; n.s., not significant; ***p < 0.001 and ****p < 0.0001 vs. cells treated with LPS only.

Ag@Se-S and Ag@Se-P demonstrated a 60% inhibition at all nanoparticle concentrations. Additionally, the measurement of cytokine production also showed that both sample treatments had a reducing influence on the amount of IL-1 and IL-6 produced by LPS-stimulated RAW 264.7 cells (Figure 8). Additionally, our findings demonstrate that both substances decreased the RAW 264.7 cells’ production of the inflammatory proteins and genes *PI3K* and *NFkB*, which are mediated by LPS (Figures 10, 11). As a result, it was hypothesized that Ag@Se-S and Ag@Se-P nanoparticles and seed and plant extracts have anti-inflammatory effects by preventing the release of pro-inflammatory mediators by LPS-stimulated RAW 264.7 cells. Previous investigations by Kim et al. (2012) and Davaatseren et al. (2014) found that allyl isothiocyanate (AITC) could inhibit LPS-induced iNOS expression *in vitro* and reduce iNOS expression during colitis *in vivo*. As a result, the delay in TNF production and iNOS expression was the primary cause of the inhibition of AITC on NO production seen in our investigation. In addition, LPS combined with nanoparticles should have a synergistic anti-inflammatory effect on RAW 264.7 macrophage cells. In a related investigation, Wang et al. (2010) showed that LBP extract could reduce iNOS expression as well as LPS-induced NO, TNF-α, and IL-6 production. Additionally, LBP’s antioxidative activity may potentially help reduce NO generation.

RAW 264.7 cells were pretreated with PA seed and plant extracts for 24 h, followed by LPS (1 μg/mL) stimulation for 18 h. The expression of phosphoinositide 3 kinase (PI3K) and nuclear factor kappa B (NFkB) was analyzed by qRT-PCR (*n* = 3). Values are represented as the mean ± SD. Statistical significance was calculated using the *t*-test and one-way ANOVA. *p* < 0.05 vs. cells treated with media only; n.s., not significant; ****p < 0.0001 vs. cells treated with LPS only.

Cells were pretreated with PA seed and plant extracts for 24 h, followed by LPS (1 μg/mL) stimulation for 18 h. Whole-cell lysates were subjected to Western blotting (a). Beta-actin was used as the internal control. The relative band intensity of phosphorylated target protein to total target protein was analyzed using ImageJ (b) (*n* = 3). Values are represented as the mean ± SD. Statistical significance was calculated using the *t*-test and one-way ANOVA. *p* < 0.05 vs. cells treated with media only; n.s., not significant; ****p < 0.0001 vs. cells treated with LPS only.

## Conclusion

Bimetallic Ag@Se-P and Ag@Se-S nanoparticles were successfully synthesized using *P. aculeata* aerial parts and seed extracts as a biogenic, facile, eco-friendly, and fast process for nanoparticle production. Different characterization techniques, such as TEM, FT-IR, SEM, EDS, and XRD, were used to study the physical and chemical properties of bimetallic nanoparticles. The resultant data confirmed that the synthesized materials are on the nanoscale, with cluster irregular spherical shapes and mean particle sizes of 21.4 and 16.95 nm for Ag@Se-P and Ag@Se-S, respectively. Additionally, the EDS results confirmed that the synthesized material was composed of Ag, Se, C, and O elements. Therefore, P. *aculeata* may have applications as a nanoparticle’s bioactive molecules and enhance the biological activities of the synthesized nanoparticles to reduce changes in physicochemical property. Additionally, during the evaluation of nanoparticles, Ag@Se-S and Ag@Se-P caused complete protection against LPS-induced inflammation in murine macrophage cell lines. To get P. *aculeata*-Ag@Se-NPs that offer excellent antioxidant and anti-inflammatory abilities, the limitation of ABE capping efficacy on the nanoparticle surface should be resolved by the optimization of the synthesis technique in future investigations. Therefore, future investigations should be conducted to explore the effects of biosynthesized particles on physical characteristics and biological tissue exposures. Thus, Ag@Se can be used as a therapeutic agent in improving the antioxidant system and reducing tissue toxicity.

## Data Availability

The datasets presented in this study can be found in online repositories. The names of the repository/repositories and accession number(s) can be found in the article/Supplementary Material.

## Abbreviations

ABTS, 2,2′-azino-bis (3-ethylbenzothiazoline-6-sulfonic acid); cDNA, copy DNA; EDS, energy-dispersive X-ray spectroscopy; ELISA, enzyme-linked immunosorbent assay; DPPH, 2,2-diphenyl-1-picrylhydrazyl; EMEM, Eagle’s minimum essential medium; FBS, fetal bovine serum; FT-IR, Fourier-transform infrared spectroscopy; HR-TEM, high-resolution transmission electron microscopy; IL-1β, interleukin-1β; IL-6, interleukin-6; iNOS, inducible nitric oxide synthase; LPS, lipopolysaccharides; mRNA, messenger ribonucleic acid MTT, 3-(4,5-dimethyl thiazol-2-yl)-2,5-diphenyl tetrazolium bromide; NFkB, nuclear factor kappa light-chain enhancer of activated B cells; PAGE, polyacrylamide gel electrophoresis; PBS, phosphate-buffered saline; PI3K, phosphoinositide 3-kinase; RT-PCR, reverse transcription-polymerase chain reaction; SEM, scanning electron microscopy; TCA, trichloroacetic acid; TNF-α, tumor necrosis factor-alpha; XRD, X-ray diffraction spectroscopy.
